# Effects of Vacuum Phenomenon on Cage Subsidence and Fusion Outcomes in Oblique Lumbar Interbody Fusion: A Cohort Study

**DOI:** 10.3390/jcm13237036

**Published:** 2024-11-21

**Authors:** Jae-Hyuk Yang, Kun-Joon Lee, Seung-Yup Lee, In-Hee Kim, Sang Yun Seok, Hansongi Suh, Hyung Rae Lee

**Affiliations:** 1 Department of Orthopedic Surgery, Korea University Anam Hospital, Seoul 02708, Republic of Korea; kuspine@korea.ac.kr (J.-H.Y.); yup1019@naver.com (S.-Y.L.); 2 College of Medicine, Korea University, Seoul 02841, Republic of Korea; slcjohn@daum.net; 3 National Police Hospital, Seoul 05715, Republic of Korea; soaringss@naver.com; 4 Department of Orthopedic Surgery, Daejeon Eulji University Hospital, Daejeon 35233, Republic of Korea; oper251@hanmail.net; 5 Department of Business Administration, Yonsei University School of Business, Seoul 03722, Republic of Korea; hansongi.suh@gmail.com

**Keywords:** vacuum phenomenon, cage subsidence, OLIF, lumbar degenerative disease, endplate sclerosis, spine surgery

## Abstract

**Background***:* Oblique lumbar interbody fusion (OLIF) is a minimally invasive technique used to manage degenerative lumbar conditions. The presence of vacuum phenomenon (VP) and associated endplate sclerosis may increase the risk of cage subsidence. This study evaluated the relationship between VP grade, endplate sclerosis, and subsidence in OLIF. **Methods**: This retrospective cohort study included 165 patients who underwent a single-level OLIF for lumbar stenosis. Patients were stratified into VP grades (0–3) based on preoperative computed tomography scans. Disc height, endplate sclerosis, and cage subsidence were radiologically assessed. Clinical outcomes, including back and leg pain visual analog scale, Oswestry Disability Index, and EuroQol-5 Dimension, were measured preoperatively and at follow-up. **Results**: High VP grades were associated with low preoperative disc height and increased endplate sclerosis. Although no significant differences in clinical outcomes or final fusion rates across VP grades were observed, the subsidence rate increased with VP grade, with a significant difference between VP grades 1 and 2 (*p* = 0.045) and between VP grades 2 and 3 (*p* = 0.032), indicating that subsidence rates increased as the VP grade advanced. **Conclusions**: High VP grades, particularly grades 2 and 3, may increase the risk of cage subsidence following OLIF. Therefore, VP grading may be worth considering during surgical planning to reduce the subsidence risk and improve outcomes.

## 1. Introduction

Oblique lumbar interbody fusion (OLIF) has emerged as an effective surgical technique for managing various spinal pathologies, including lumbar degenerative diseases, spinal deformities, trauma, and neoplasms [1,2,3]. Introduced to provide a safe anterior approach to the lumbar disc, OLIF offers a unique route between the aorta and psoas muscle, avoiding direct traversal through the psoas [4]. This method reduces the risk of lumbar plexus injury. Additionally, OLIF facilitates direct visualization of critical structures such as the sensory nerves, ureter, major blood vessels, lymphatics, and sympathetic trunk, contributing to its safety profile [5,6]. These advantages have positioned OLIF as a valuable and relatively safe option for interbody fusion procedures.

The presence of vacuum phenomenon (VP) is often regarded as a favorable indicator of spinal fusion, particularly when considering OLIF [7,8,9,10,11]. VP, which represents the accumulation of gas within the disc space, is typically associated with segmental instability [8,9,12,13,14]. This instability frequently necessitates surgical stabilization, making fusion the appropriate intervention. Because VP is a marker of instability, OLIF, with its minimally invasive approach and ability to restore disc height and segmental stability, is an ideal option for addressing such cases [9,10,11,14,15]. Furthermore, VP occurs in degenerated disc spaces, where the structural changes align well with the indications for OLIF [5,16].

Although VP is often considered a reliable indicator of fusion owing to its association with segmental instability, its impact on other surgical outcomes, particularly in relation to endplate sclerosis and cage subsidence, remains uncertain [17,18]. Endplate sclerosis, frequently observed in patients with VP, strengthens the endplate and supports fusion; however, it may also lead to complications such as cage subsidence [4,14,16,19,20]. This contradictory role of endplate sclerosis highlights the need for elucidating its relationship with fusion success and other surgical outcomes in OLIF.

Cage subsidence, a well-documented complication of OLIF, is typically associated with intraoperative endplate damage, osteoporosis, and other postoperative factors [3,4,16,21]. Although endplate sclerosis may protect against subsidence by increasing endplate strength, some studies have suggested that it may paradoxically increase the subsidence risk by reducing the flexibility needed to distribute mechanical load [20]. The complex relationship among VP grades, endplate sclerosis, and cage subsidence remains underexplored, and existing evidence is conflicting.

Thus, this study investigated the influence of VP presence and grading on fusion outcomes and the risk of cage subsidence in OLIF. By evaluating the relationship between VP grades, endplate sclerosis, and surgical outcomes, we aimed to provide further insights into optimizing fusion success and reducing the complications associated with OLIF.

## 2. Materials and Methods

### 2.1. Study Design and Patient Selection

This cohort study was approved by the institutional review board (IRB no. 2024AN0318, approval date: 15 July 2024). This study adhered to the ethical principles outlined in the Declaration of Helsinki and followed the Strengthening the Reporting of Observational Studies in Epidemiology (STROBE) guidelines to ensure transparency and rigor in observational research [22]. The study enrolled patients who underwent single-level OLIF during minimally invasive surgery for degenerative stenosis between July 2018 and June 2022. The patients included in the study presented with symptoms of neurogenic claudication and/or sciatica due to central stenosis or severe leg pain from foraminal stenosis. OLIF was selected for those with radiographic evidence of segmental instability or severe foraminal height loss, as well as for patients experiencing dynamic low back pain. These conditions would likely reduce the effectiveness of decompression alone. In such cases, the OLIF offered benefits by restoring stability and foraminal height, thereby enhancing symptom improvement. Patients with prior surgery on the affected segment, infections, trauma, tumors, insufficient follow-up, or incomplete medical and radiographic data were excluded. Finally, 165 patients were included and categorized by VP grade observed in the disc space at the surgical level on computed tomography (CT) scans.

### 2.2. Surgical Technique

OLIF was performed with the patient in the lateral decubitus position. Using an oblique approach, the disc space was accessed anteriorly, bypassing the psoas muscle, to minimize the risk of lumbar plexus injury [2]. The intervertebral disc was removed, and a polyetheretherketone (PEEK) interbody cage was inserted to restore the disc height and correct spinal alignment. Demineralized bone matrix was used as an interbody graft. Percutaneous pedicle screws were fixed posteriorly to provide additional stability to operated segments. All procedures were performed by the senior author to ensure consistency in surgical technique across patients.

### 2.3. Data Collection and Radiologic Assessments

**Figure 1:**
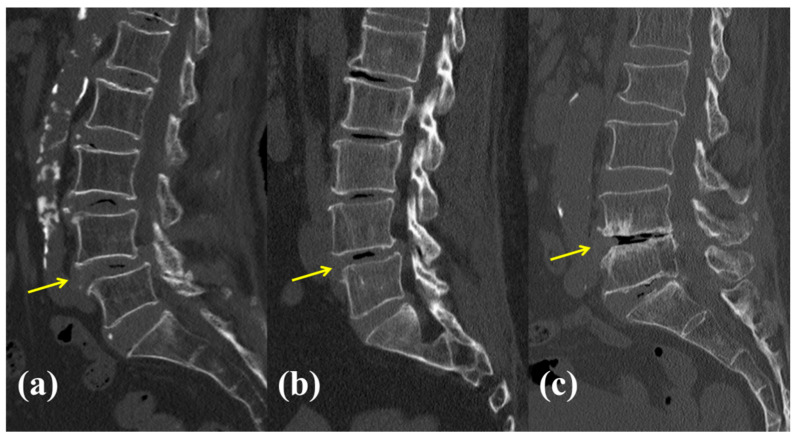
Classification of vacuum phenomenon (VP) grade based on the spatial percentage relationship between VP and the disc space. VP grade was evaluated in the L4-5 disc space (yellow arrow). Grades 1, 2, and 3 were assigned when the VP occupied < 20% (**a**), between 20% and 80% (**b**), and >80% (**c**) of the disc space, respectively.

**Figure 2:**
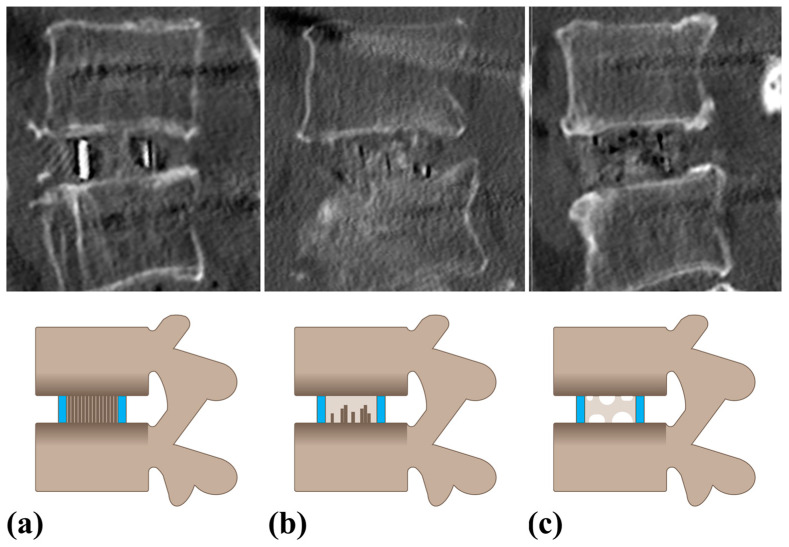
Example of grading method for evaluating fusion. (**a**) Grade I: Fused with evidence of remodeling and trabecular formation. (**b**) Grade II: Not fully remodeled or incorporated and without lucency. (**c**) Grade III: Lucency is observed at the graft site.

Demographic data including age, sex, height, weight, body mass index, bone mineral density (BMD), and underlying comorbidities, including hypertension, diabetes mellitus, smoking status, and other underlying diseases, were thoroughly reviewed from the patients’ electronic medical records. Radiological assessments included determining the location of the surgical site and evaluating segmental instability, with a particular focus on identifying isthmic spondylolisthesis (ISL) using plain dynamic lumbar lateral radiographs. VP was assessed using preoperative CT scans, and VP grades were classified into grade 0 (no VP), grade 1 (VP < 20%), grade 2 (20% ≤ VP < 80%), and grade 3 (VP ≥ 80%) (Figure 1) [12]. Additionally, endplate sclerosis was observed. Quantitative radiological measurements included anterior disc height (ADH) and posterior disc height (PDH), both of which were measured preoperatively and postoperatively in the sagittal CT plane using the Picture Archiving and Communication System (PetaVision for Clinics, 3.1, Korea University Anam Hospital, Seoul, Republic of Korea). Cage insertion angles were also measured in the axial and coronal planes using postoperative CT evaluation. The axial plane angle was defined as positive when the cage was inserted from the left anterior to right posterior direction, whereas the coronal plane angle was defined as negative when the cage was inserted from the left superior to right inferior direction. Postoperative fusion was assessed using a modified approach based on the Bridwell classification of CT scans (Figure 2) [23]. Because a PEEK cage was used, fusion was assessed both inside and outside the cage. Additionally, implant-related complications including screw violations, loosening, and breakage were assessed. The occurrence of subsidence owing to endplate damage was meticulously assessed, and the location of subsidence, whether at the upper or lower endplate, was documented.

### 2.4. Clinical Outcome Measures

Clinical outcomes were measured preoperatively and at 1 and 2 years postoperatively. The assessments included the back pain visual analog scale (VAS), leg pain VAS, Oswestry Disability Index (ODI), and EuroQol-5 Dimension (EQ-5D). The back pain VAS is a subjective scale ranging from 0 (no pain) to 10 (worst imaginable pain) and was selected to evaluate the effectiveness of fusion in reducing axial spine-related pain. The leg pain VAS, also rated from 0 to 10, specifically measures neurogenic leg pain, a common symptom in lumbar spinal stenosis. The ODI, with a scale from 0 (no disability) to 100 (complete disability), measures the extent of functional limitations and is critical for assessing improvements in mobility and daily activities. The EQ-5D evaluates overall quality of life across five domains (mobility, self-care, usual activities, pain/discomfort, and anxiety/depression), with a total score range of 5 to 25. The MCID values for each clinical outcome measure in this study are based on reference standards from lumbar spine surgery studies; for ODI, an MCID of 12.8 points reflects meaningful functional improvement, while the MCID values for back pain VAS and leg pain VAS are 1.2 and 1.6 points, respectively, indicating clinically significant pain reduction [24].

### 2.5. Statistical Analyses

Statistical analyses were conducted using the SPSS software (version 26.0; IBM Corp., Armonk, NY, USA). Continuous variables are expressed as means ± standard deviations, whereas categorical variables are presented as numbers and percentages. The normality of continuous variables was tested using the Shapiro–Wilk test. Independent t-tests were used to compare continuous variables between the VP grade groups, and chi-square or Fisher’s exact tests were used for categorical variables, where applicable. Univariate and multivariate logistic regression analyses were performed to identify the risk factors for subsidence. To compare clinical outcomes between different VP grades, repeated-measures analysis of variance was used for normally distributed continuous variables, with post hoc pairwise comparisons using Bonferroni correction. Statistical significance was set at *p* < 0.05.

## 3. Results

### 3.1. Patient Characteristics

**Figure 3:**
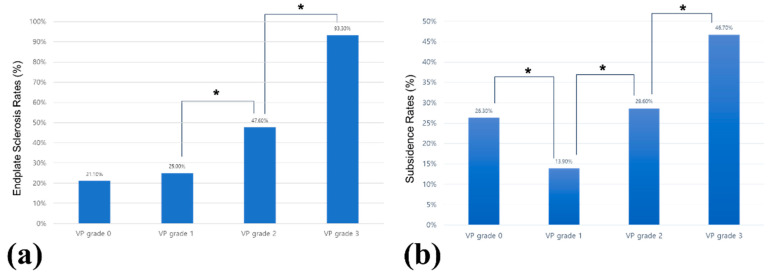
Comparison of endplate sclerosis and subsidence rates across vacuum phenomenon (VP) grades. (**a**) Endplate sclerosis was more frequently observed with increasing VP grades, with significant differences between grades 1 and 2 and between grades 2 and 3. (**b**) Subsidence rates were lowest in VP grade 1. Significant differences were observed between grades 1 and 0 and between grades 1 and 2, with subsidence rates increasing progressively from grade 1 to grade 3. The asterisk (*) indicates a statistically significant difference (*p* < 0.05).

**Figure 4:**
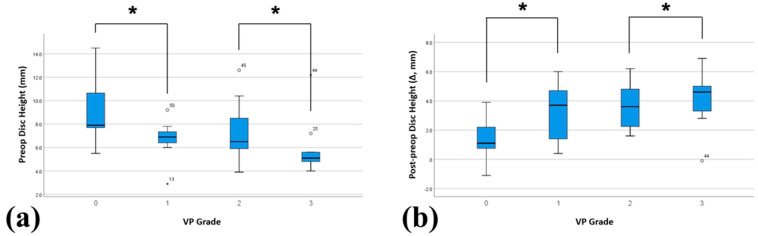
Comparison of preoperative disc height and disc height restoration across vacuum phenomenon (VP) grades. (**a**) Preoperative disc height comparison by VP grades. A significant difference was observed between grades 0 and 1 and between grades 2 and 3, with lower disc height observed in higher VP grades. (**b**) Postoperative disc height restoration (postoperative disc height–preoperative disc height) also showed significant differences between grades 0 and 1 and between grades 2 and 3, with greater restoration occurring in higher VP grades. The asterisk (*) indicates a statistically significant difference (*p* < 0.05).

The preoperative characteristics of the patients stratified by VP grade are summarized in Table 1. Patients with high VP grades had significantly small preoperative disc heights. The ADH and PDH decreased as the VP grade increased (*p* = 0.027 for ADH, *p* = 0.002 for PDH). Additionally, patients with high VP grades likely had endplate sclerosis, with a notable increase in the prevalence of grade 3 VP (*p* < 0.001) (Figure 3a). Analysis of the average disc height showed that as the VP grade increased, the disc height decreased (Figure 4a). No significant difference in the incidence of ISL was observed between the VP grade groups (*p* = 0.343). Additionally, no significant differences were observed in basic demographics, such as age, sex ratio, height, weight, and BMD, across the groups.

### 3.2. Surgical Outcomes

Surgical outcomes stratified by VP grade are shown in Table 2. The differences in disc height observed preoperatively between the VP grades were no longer significant postoperatively. Both anterior and posterior disc heights were restored to similar levels across all VP grades postoperatively. However, the degree of height restoration (postoperative height minus preoperative height) was significantly greater in patients with higher VP grades, indicating that patients with a lower preoperative disc height experienced a more substantial increase following cage insertion (*p* = 0.049 for anterior height and *p* = 0.009 for posterior height) (Figure 4b). The cage insertion angles in both the axial and coronal planes did not indicate significant differences across the VP grades (*p* = 0.733 and *p* = 0.175, respectively). When the fusion outcomes were evaluated, no significant differences were observed among VP grades (*p* = 0.855).

### 3.3. Clinical Outcomes

**Figure 5:**
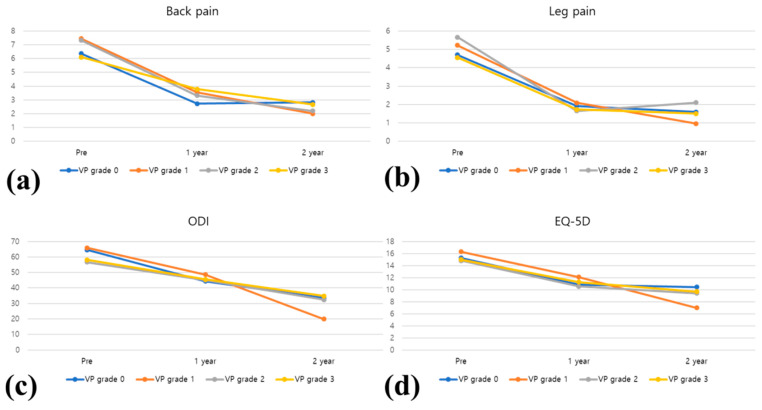
Results of patient-reported outcomes, including back pain, leg pain, Oswestry Disability Index (ODI), and EuroQol-5 Dimension (EQ-5D). (**a**) Back pain improvement. (**b**) Leg pain improvement. (**c**) ODI scores. (**d**) EQ-5D scores. Although VP grade 1 showed the most improvement, no significant difference was observed between VP grade subgroups across different time points.

The clinical outcomes, including back pain VAS, leg pain VAS, ODI, and EQ-5D, in every group generally met the MCID thresholds after fusion, indicating meaningful clinical improvement [25]. However, no significant differences in the clinical outcomes, including back pain VAS, leg pain VAS, ODI, and EQ-5D, were observed among VP grades. Although VP grade 1 group showed the greatest improvement, the differences between the VP subgroups were not significant at any postoperative time point (Figure 5). 

### 3.4. Complications

**Figure 6:**
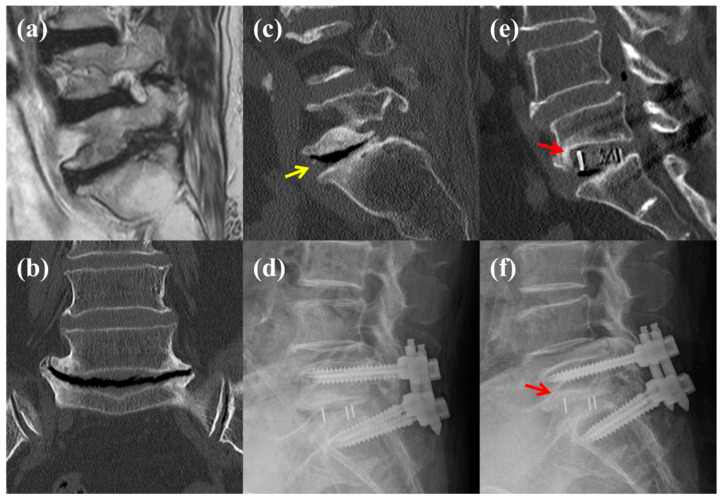
A representative case of subsidence following oblique lateral interbody fusion surgery in a patient with vacuum phenomenon (VP) grade 3 at the L5–S1 level. The patient presented with severe radiculopathy in the right L5 dermatome, causing claudication and radiating pain. Conservative treatment was unsuccessful, and surgery was performed. (**a**) Sagittal magnetic resonance imaging revealed right L5 nerve root compression owing to L5–S1 foraminal stenosis. (**b**) Coronal computed tomography (CT) view showed collapse across the entire L5–S1 disc, bilateral disc space spurring, and a prominent VP. (**c**) Sagittal CT view demonstrated disc collapse, endplate sclerosis, and VP grade 3 at the L5–S1 (yellow arrow). (**d**) Immediate postoperative plain lateral radiograph confirmed the disc space height restoration because of the cage. (**e**,**f**) At 2 months postoperatively, subsidence was observed (red arrow), with CT sagittal and plain lateral radiographs revealing L5 endplate damage and cage subsidence into the L5 vertebral body.

Implant-related complications, including pedicle wall violation, screw loosening, and rod and screw breakage, did not differ significantly among the VP grades (Table 3). Similarly, vessel injury and reoperation rates were not significantly different between the groups. However, the subsidence rates were clearly distinct between the VP grades. The incidence of subsidence was lowest in VP grade 1 (13.9%) and increased progressively with higher VP grades, reaching 46.7% in VP grade 3 (*p* = 0.031) (Figure 3b). When subsidence location was analyzed, a pattern emerged wherein subsidence predominantly occurred at the upper endplate in VP grades 0 and 1, whereas it was more common at the lower endplate in VP grades 2 and 3 (*p* = 0.014). A representative case of subsidence in a patient with VP grade 3 is illustrated in Figure 6, where significant endplate damage and cage subsidence were observed postoperatively. This patient, who initially presented with severe radiculopathy and claudication, underwent OLIF surgery with successful disc height restoration, although subsidence subsequently occurred, as confirmed by CT and plain radiographs at the 2-month follow-up.

### 3.5. Regression Analysis of Subsidence Risk Factors

The results of the univariate and multivariate analyses assessing the factors affecting subsidence are presented in Table 4. In the univariate analysis, age (*p* = 0.003) and endplate sclerosis (*p* < 0.001) were significant predictors of subsidence. In the multivariate analysis, both age (*p* = 0.015) and endplate sclerosis (*p* < 0.001) remained associated with an increased subsidence risk. The VP grade showed a trend toward significance in the univariate analysis (*p* = 0.056), although this did not remain significant in the multivariate model.

Additional analysis of patients with VP (n = 108) confirmed that VP grade was a significant factor affecting subsidence when patients with VP grade 0 were excluded. As shown in Table 5, both VP grade (*p* = 0.005) and endplate sclerosis (*p* = 0.019) were significant risk factors for subsidence in the univariate analysis. However, in the multivariate analysis, VP grade (*p* = 0.014) was the only significant predictor of subsidence. Age, BMD, and the cage insertion angle were not significant predictors in the multivariate model.

## 4. Discussion

This study investigated the relationship between VP grade, endplate sclerosis, and surgical outcomes, particularly cage subsidence, in patients undergoing OLIF. Our findings indicate that high VP grades were associated with low preoperative disc height, increased endplate sclerosis, and significantly high subsidence rates. These results elucidate the complex interactions between disc degeneration, endplate conditions, and fusion outcomes.

One key observation was that patients with high VP grades had significantly low preoperative disc heights (Table 1 and Figure 4a). This reduction in disc height is likely caused by chronic degenerative changes, in which severe disc collapse and facet joint stiffening occured in unstable segments [7,11,12,13,19,26]. Such instability may arise from conditions such as spondylolysis or degenerative changes. Consequently, despite efforts to restore the disc height during OLIF in patients with advanced VP grades, stiffened facets and contracted disc space could limit the amount of height restoration that can be achieved by placing increased mechanical stress on the inserted cage [4,5,16]. This added stress could explain the higher incidence of subsidence in patients with higher VP grades, as the cage must bear more of the load, particularly when endplate sclerosis is present. Additionally, the severely reduced disc height in these patients increases the likelihood of endplate damage during the disc preparation process, making the damaged endplate more susceptible to failure under cage pressure [2]. These findings underscore the need for meticulous endplate preparation to minimize the subsidence risk in patients with severely collapsed discs and advanced VP grades.

Endplate sclerosis is considered a favorable factor for preventing cage subsidence because it indicates increased bone density and structural integrity [4,16]. However, our results suggest that when combined with severe VP, endplate sclerosis increased the subsidence risk. In the regression analysis of the entire study cohort, endplate sclerosis was identified as the most significant predictor of subsidence (Table 4). Previous studies have reported mixed findings regarding the role of endplate sclerosis in subsidence. Chung et al. [27] identified no significant correlation between endplate sclerosis and subsidence, whereas other studies have reported that endplate sclerosis negatively affects subsidence, which is consistent with our findings [4,20]. Although sclerosis may offer initial structural advantages, its excessive stiffness can hinder proper height restoration, forcing the cage to bear excessive loads, ultimately leading to subsidence [21]. This was particularly evident in patients with VP grades 2 and 3, in whom subsidence occurred more frequently despite the presence of endplate sclerosis. However, these results need to be interpreted with caution, as patients with higher VP grades are more likely to have endplate sclerosis and lower disc heights, which introduces a potential bias. Interestingly, our study showed that patients with VP grade 1 exhibited the lowest subsidence rates (Figure 3b), suggesting that mild VP and moderate endplate sclerosis may have protective effects by allowing adequate height restoration without overburdening the cage [16]. By contrast, in patients with higher VP grades and more severe sclerosis, the increased mechanical load on the cage coupled with severely reduced disc height may increase the likelihood of subsidence. Therefore, although endplate sclerosis can be beneficial in certain cases, it may be a risk factor in patients with more severe VP.

The regression analysis in Table 4 reveals that in both the univariate and multivariate analyses, age and endplate sclerosis were significant predictors of subsidence, whereas VP grades were not. One possible explanation for the absence of VP grade as a significant factor could be the subsidence pattern observed in our study; VP grade 1 had the lowest incidence of subsidence, even lower than VP grade 0 (Figure 3b). Thus, VP grade 1 might exert a protective effect against subsidence compared with no presence of VP. We hypothesized that the mild presence of VP (grade 1) would allow for better load distribution and less mechanical stress on the cage, thus reducing the likelihood of subsidence. Furthermore, disc space instability associated with VP grade 1, coupled with mild sclerosis and other degenerative changes, may improve bone quality at the cage–endplate interface [4,7,16,19]. Mild sclerosis strengthens the endplate where the cage contacts it, potentially contributing to the protective effect observed in this subgroup [16,28]. However, as VP grades increase to 2 and 3, the disc height becomes significantly reduced, leading to greater height restoration during surgery [21,29,30]. This increased disc height restoration elevates the mechanical load on the endplate, which, if the fusion does not occur at the optimal time, can compromise endplate integrity and lead to failure. Poor endplate integrity subsequently affects cage stability, increasing the risk of subsidence. Moreover, reduced endplate integrity in these higher VP grades can diminish the support structure for the cage, impairing stability and increasing mechanical stress across the endplate–cage interface. This interplay between diminished integrity, increased mechanical load, and reduced stability highlights the intricate role that VP grades play in surgical outcomes.

Moreover, the risk of iatrogenic damage during endplate preparation may increase as the VP grades increase to 2 and 3 because the disc space becomes smaller and more challenging to work on [20,29]. This could explain the conflicting results regarding the role of VP in the subsidence risk reported in previous studies. Some studies have suggested that VP may protect against subsidence [17,31], whereas others have indicated that VP increases the subsidence risk [4,16]. The complex interaction between VP, disc height, and endplate condition may underlie these varying findings. The significance of our study lies in its relatively large cohort in which we analyzed subsidence risk according to specific VP grades. Moreover, VP grade 1 provided a more favorable outcome for subsidence than VP grade 0, supporting the hypothesis of a protective effect at this stage [16]. However, as the VP grades increased to 2 and 3, the subsidence risk significantly increased. This underscores the importance of considering VP grade when planning surgical interventions, particularly in terms of preventing subsidence. These findings were even more evident in the subgroup analysis of patients with VP (Table 5). When VP grade 0 was excluded, the VP grade emerged as the sole significant predictor of subsidence in the multivariate analysis (*p* = 0.014). This further supports the idea that although mild VP (grade 1) may confer some protection against subsidence, higher VP grades (grades 2 and 3) present a substantial risk. Endplate sclerosis, although significant in the univariate analysis, did not remain significant in the multivariate model, further emphasizing the critical role of VP grade in determining subsidence risk.

Age was identified as a significant risk factor for subsidence in the univariate analysis, which aligns with findings in the existing literature on age-related degeneration and its impact on spinal surgery outcomes [20,30,32]. Older patients often exhibit more advanced degenerative changes, including endplate sclerosis, which can compromise the structural integrity of the endplate during surgery, leading to a higher subsidence risk [32]. Interestingly, BMD was not a significant predictor of subsidence. One reason for this could be the limitation of DEXA scans, which provide an average bone density measurement across the lumbar spine (L1–L5), rather than being specific to the surgical level. More precise measurements of bone density directly at the surgical site, such as Hounsfield units (HU) obtained from CT scans near the endplate, may offer a better predictive value for subsidence [25]. Future studies should investigate this further to understand the role of localized bone density in surgical outcomes.

Our findings have surgical implications, particularly for identifying patients at a higher subsidence risk based on VP grades and endplate conditions. Surgeons should be cautious in cases of advanced VP grades (grades 2 and 3) because these patients have an increased subsidence risk. One strategy may be to avoid aggressive disc height restoration, which can place excessive stress on the endplate, particularly if it is sclerotic [20,21,28,32]. Moreover, careful intraoperative endplate preparation is essential to minimize iatrogenic damage in these patients with compromised disc spaces [20]. The protective effect observed in patients with VP grade 1 suggests that for mild instability and moderate sclerosis, OLIF remains a viable and safe option, as adequate height restoration can be achieved without significantly increasing the subsidence risk.

This study had certain limitations. First, owing to the retrospective study design, selection bias and potential confounders that could not be controlled for were inherent. Second, although our follow-up period was sufficient to capture early- and mid-term outcomes, long-term data are necessary to fully understand the impact of subsidence on fusion success and quality of life. Third, our assessment of BMD using DEXA scans may have limited the accuracy of our analysis on the role of BMD in subsidence. As mentioned above, measuring BMD directly at the surgical level or using CT-based HU could provide more specific and meaningful insights into the relationship between bone quality and subsidence risk [25]. Future studies should consider the use of these techniques to improve the precision of BMD-related findings. Although no significant differences in back pain, leg pain, or other clinical outcomes were observed among VP grades in our study, this could be attributable to the fact that subsidence in these patients did not lead to complete collapse, possibly owing to the use of BMP-2 or other mitigating factors. Lastly, the fusion outcomes did not show major differences, which may explain the lack of clinical differences. However, this should be interpreted with caution because our results may reflect a moderate scenario in which subsidence did not progress to a sufficiently severe stage to affect clinical outcomes. Subsidence remains an undesirable complication from a surgeon’s perspective, and its potential to influence clinical outcomes has been well documented in other studies. Although our findings did not demonstrate a significant clinical impact in the short to medium term, the subsidence risk should not be overlooked. Considering that subsidence has been linked to worse long-term outcomes in other studies, our study provides valuable insights into the factors contributing to subsidence, particularly VP grade and endplate sclerosis, and offers guidance for improving patient care.

## 5. Conclusions

Higher VP grades were associated with an increased subsidence risk, particularly in patients with severe endplate sclerosis and reduced disc height. Although VP grade 1 may provide some protective effects, advanced VP grades significantly increased mechanical stress on the cage, leading to a higher likelihood of subsidence. Careful surgical planning and endplate preparation are essential to minimize complications, particularly in patients with advanced VP and endplate sclerosis.

## Figures and Tables

**Table 1:** Comparison of preoperative characteristics between groups stratified by VP grade.

	VP Gr 0 (n = 57)	VP Gr 1 (n = 36)	VP Gr 2 (n = 42)	VP Gr 3 (n = 30)	*p*-Value
Age	71.2 ± 9.8	70.5 ± 8.0	72.4 ± 5.7	72.3 ± 6.4	0.918
Number of females, n	29 (50.9%)	21 (58.3)	25 (59.5%)	19 (63.3%)	0.111
Height, cm	159.2 ± 5.2	155.9 ± 8.5	156.8 ± 6.8	155.8 ± 5.7	0.437
Weight, kg	64.5 ± 11.7	61.4 ± 9.7	62.3 ± 11.0	61.5 ± 11.7	0.285
BMD, T-score	−1.7 ± 1.9	−1.5 ± 1.7	−1.4 ± 2.7	−1.2 ± 1.5	0.326
HTN, n	15 (26.3%)	12 (33.3%)	11 (26.2%)	10 (33.3%)	0.688
DM, n	4 (7.0%)	13 (36.1%)	8 (19.0%)	5 (16.7%)	0.637
Smoking, n	5 (8.8%)	11 (30.6%)	7 (16.7%)	3 (10.0%)	0.189
Psychotic disease, n	2 (3.5%)	1 (2.8%)	5 (11.9%)	3 (10.0%)	0.243
Liver disease, n	3 (5.3%)	3 (8.3%)	6 (14.3%)	2 (6.7%)	0.438
Pulmonary disease, n	3 (5.3%)	5 (13.9%)	7 (16.7%)	2 (6.7%)	0.227
Location					0.718
L1-2	1	-	-	-	
L2-3	3	2	2	1	
L3-4	5	4	3	3	
L4-5	41	25	35	22	
L5-S1	7	5	2	4	
Isthmic spondylolisthesis	1 (1.8%)	3 (8.3%)	2 (4.8%)	3 (10.0%)	0.343
Preop ADH, mm	9.7 ± 2.5	7.9 ± 2.5	7.7 ± 2.6	6.4 ± 2.4	0.027 *
Preop PDH, mm	6.3 ± 2.1	5.6 ± 1.0	5.1 ± 1.5	3.8 ± 1.5	0.002 *
Endplate sclerosis, n	12 (21.1%)	9 (25.0%)	20 (47.6%)	28 (93.3%)	<0.001 *

VP, vacuum phenomenon; BMD, bone mineral density; HTN, hypertension; DM, diabetes mellitus; ADH, anterior disc height; PDH, posterior disc height * *p*-value < 0.05.

**Table 2:** Comparison of surgical outcomes between groups stratified by VP grade.

	VP Gr 0 (n = 57)	VP Gr 1 (n = 36)	VP Gr 2 (n = 42)	VP Gr 3 (n = 30)	*p*-Value
Postop ADH	11.1 ± 2.3	11.5 ± 1.8	11.4 ± 1.7	10.5 ± 2.7	0.325
Postop PDH	7.1 ± 2.0	9.3 ± 2.3	8.5 ± 1.8	9.1 ± 0.9	0.594
ΔADH (postop–preop)	1.4 ± 1.7	3.6 ± 3.1	3.7 ± 1.9	4.1 ± 1.2	0.049 *
ΔPDH (postop–preop)	0.8 ± 1.5	3.7 ± 2.2	3.4 ± 1.5	5.3 ± 2.0	0.009 *
Cage insertion angle, ° (axial plane)	3.2 ± 3.5	2.0 ± 2.4	3.6 ± 5.0	1.2 ± 7.6	0.733
Cage insertion angle, ° (coronal plane)	−1.3 ± 2.6	−2.2 ± 1.9	−2.1 ± 2.4	−2.7 ± 2.4	0.175
Bridwell fusion grade					0.855
I	11 (19.3%)	9 (25.0%)	6 (14.6%)	8 (26.7%)	
II	24 (42.1%)	13 (36.1%)	20 (48.8%)	11 (36.7%)	
III	22 (38.6%)	14 (38.9%)	15 (36.6%)	11 (36.7%)	

VP, vacuum phenomenon; ADH, anterior disc height; PDH, posterior disc height * *p*-value < 0.05.

**Table 3:** Comparison of surgical complications between groups stratified by VP grade.

	VP Gr 0 (n = 57)	VP Gr 1 (n = 36)	VP Gr 2 (n = 42)	VP Gr 3 (n = 30)	*p*-Value
Implant related complications					
Pedicle wall violation	2 (3.5%)	1 (2.8%)	2 (4.8%)	4 (13.3%)	0.206
Screw loosening	5 (8.8%)	1 (2.8%)	3 (7.1%)	3 (10.0%)	0.659
Rod and screw breakage	1 (1.8%)	0 (0.0%)	1 (2.4%)	2 (6.7%)	0.350
Vessel injury	0 (0.0%)	0 (0.0%)	1 (2.4%)	1 (3.3%)	0.432
Subsidence	15 (26.3%)	5 (13.9%)	12 (28.6%)	14 (46.7%)	0.031 *
Subsidence location					0.014 *
Upper endplate	9 (15.8%)	3 (8.3%)	3 (7.1%)	3 (10.0%)	
Lower endplate	6 (10.5%)	2 (5.6%)	9 (21.4%)	11 (36.7%)	
Reoperation	0 (0.0%)	0 (0.0%)	1 (2.4%)	2 (6.7%)	0.125

VP, vacuum phenomenon * *p*-value < 0.05.

**Table 4:** Univariate and multivariate analysis of factors affecting subsidence after single-level OLIF.

	Beta	S.E.	Wald	Exp (B)	Lower CI	Upper CI	*p*-Value
Univariate analysis							
Sex	0.05	0.39	0.12	1.05	0.47	2.24	0.908
Age	0.07	0.02	2.93	1.07	1.03	1.13	0.003 *
BMD	−0.03	0.12	−0.31	0.96	0.73	1.23	0.759
Preop ADH	−0.33	0.31	1.16	0.72	0.39	1.32	0.282
Preop PDH	0.25	0.35	0.51	1.28	0.65	2.53	0.481
Cage insertion angle (Axial)	0.14	0.12	1.34	1.15	0.91	1.47	0.255
Cage insertion angle (Coronal)	0.25	0.31	0.65	1.28	0.7	2.33	0.423
Endplate sclerosis	1.48	0.37	4	4.41	2.16	9.32	<0.001 *
VP grade	0.29	0.15	1.91	1.35	1	1.84	0.056
Multivariate analysis							
(Intercept)	−6.31	1.96	−3.21	0	0	0.07	0.001
Age	0.0645	0.02	2.43	1.07	1.01	1.13	0.015 *
Endplate sclerosis	1.37	0.37	3.65	3.97	1.92	8.51	<0.001 *

Residual deviance/df = 171.9/162 = 1.06, pseudo-R2 = 0.19 (Nagelkerke); OLIF, oblique lateral interbody fusion; S.E., standard error; CI, confidence interval; BMD, bone mineral density; ADH, anterior disc height; PDH, posterior disc height; VP, vacuum phenomenon * *p*-value < 0.05.

**Table 5:** Univariate and multivariate analysis of factors affecting subsidence after single-level OLIF in patients with VP (n = 108).

	Beta	S.E.	Wald	Exp (B)	Lower CI	Upper CI	*p*-Value
Univariate analysis							
Sex	1.13	0.53	2.12	3.09	1.08	8.9	0.064
Age	−0.02	0.03	−0.56	0.98	0.92	1.05	0.573
BMD	−0.25	0.17	−1.46	0.78	0.53	1.06	0.145
Preop ADH	−1.10	1.09	−1.01	0.33	0.02	1.99	0.314
Preop PDH	0.61	0.58	1.05	1.84	0.59	5.85	0.294
Cage Insertion Angle (Axial)	0.67	0.68	0.99	1.95	0.51	7.57	0.324
Cage Insertion Angle (Coronal)	0.25	0.28	−0.55	0.86	0.49	1.5	0.585
Endplate sclerosis	1.08	0.46	2.35	2.93	1.23	7.48	0.019 *
VP grade	0.84	0.30	2.83	2.31	1.32	4.24	0.005 *
Multivariate analysis							
(Intercept)	−2.44	0.75	−3.25	0.09	0.02	0.35	0.001 *
VP grade	0.86	0.35	2.47	2.36	1.22	4.83	0.014 *

Residual deviance/df = 98.9/82 = 1.21, pseudo-R2 = 0.388 (Nagelkerke); BMD, bone mineral density; S.E., standard error; CI, confidence interval; Preop ADH, preoperative anterior disc height; Preop PDH, preoperative posterior disc height; VP, vacuum phenomenon * *p*-value < 0.05.

## Data Availability

The datasets used and/or analyzed in the current study are available from the corresponding author upon reasonable request.
